# Education Differences in Older Adults' Performance on Online Assessments of Inductive Reasoning and Verbal Memory

**DOI:** 10.1093/geroni/igab046.2644

**Published:** 2021-12-17

**Authors:** Grace Caskie, Abigail Voelkner

**Affiliations:** 1 Lehigh University, Bethlehem, Pennsylvania, United States; 2 Lehigh University, Easton, Pennsylvania, United States

## Abstract

Paper-and-pencil measures of inductive reasoning and verbal memory administered in-person are well-established methods for measuring cognitive ability in adults. However, given recent increases in the use of online surveys, particularly during the COVID-19 pandemic when in-person research with older adults became difficult, we investigated whether these cognitive measures could be administered effectively online and whether older adults’ performance on these measures of inductive reasoning and verbal memory might differ by education level. Data were collected online between mid-May and mid-June of 2020 from 292 individuals aged 66-90 years (M=69.1, SD=3.3). The sample was primarily White (91%) and had more women (62%) than men; 83 participants had a graduate-level education (master’s/doctoral degree), 101 had an associate’s or bachelor’s degree, and 108 had less than an associate’s degree. Three measures of inductive reasoning (Number Series, Letter Sets, and Word Series) and two measures of verbal memory (Immediate Recall and Delayed Recall of a list of 20 words) were completed by participants on an online platform. One-way MANOVA found a significant main effect for education group on the inductive reasoning measures (Wilks’ lambda=.93, p=.001). However, follow-up univariate ANOVAs indicated significant differences by education group only for Number Series, with Tukey post hoc tests showing that the graduate-level and college-degree groups performed significantly better than the group with less than an associate’s degree. Factorial repeated-measures ANOVA found a significant decline between immediate and delayed recall (p<.001) and that this difference varied by education group (p=.003). Implications of these findings will be discussed.

